# 

**Published:** 1905-12-16

**Authors:** 


### NOTICE TO CORRESPONDENTS.

All MSS., Letters, Books for Review, and other matters intended for the Editor should be addressed The Editor, "The Hospital," The Hospital Buildings, 28 & 29 Southampton Street, Strand, W.C.

The Editor cannot undertake to return rejected MS., even when accompanied by stamped directed envelope.

**Notice to Advertisers and Subscribers.**

All Advertisements, Orders for Copies of The Hospital, and Business Communications should be addressed to **THE MANAGER (Not to the Editor),** The Hospital, 28 & 29 Southampton Street, Strand, London, W.C.

The Cost of Subscription, post free, is at follows:—Per week, 3½d.; per quarter, 3s. 3d.; per half-year, 6s. 6d.; per annum, 13s. Foreign and Colonial:—Per annum, 19s. 6d., payable, in all cases, in advance.

**NOTICE.—Vol. XXXVIII. of "The Hospital" April to September 1905), in handsome cloth binding, will shortly be on sale at the office of this Journal, price 10s. 6d. Cases for binding the Numbers contained in this Volume 2s. Index to Vol. XXXVIII., 3½d., post free.**

**The Monthly Part for December is now ready. Price 1s., by post, 1s. 3d.**

### MEDICAL APPOINTMENTS.

**Archibald Craig Amy.** M.B., Ch.B.Glasg., Resident House Physician at the Queen's Jubilee Hospital, London, S.W. **Alfred Coleridge,** L.R.C.P.Lond., M.R.C.S., Medical Officer to the Bristol Dispensary. **Franklin Cox,** L.R.C.P.Lond., M.R.C.S., Medical Officer and Public Vaccinator for the Radstock District by the Clutton (Somerset) Board of Guardians. **Charles L'Oste Miall,** L.R.C.P.Lond., M.R.C.S., Medical Officer and Public Vaccinator for the Paulton District by the Clutton (Somerset) Board of Guardians. **George Harper Pearce,** D.P.H.Cantab., L.R.C.P.&S.Edin., L.F.P.S.Glasg., re-appointed Medical Officer of Health to the Darton Urban District Council. **George Samuel Pollard,** L.R.C.P.Edin., M.R.C.S., Medical Officer and Public Vaccinator for the Midsomer Norton District by the Clutton (Somerset) Board of Guardians. **Arthur William Cecil Richardson,** M.B. Lond., Medical Officer to the Bristol Dispensary. **Alfred Tom Rimell,** M.D.Durh., Medical Officer and Public Vaccinator to the Third, Fourth, and Tenth Districts of the Tendring Union, Essex. **Douglas W. Sibbald,** M.B., Ch.B.Edin., Resident House Surgeon at the Queen's Jubilee Hospital, London, S.W. **Sydney Stephenson,** M.B., C.M., F.R.C.S.Edin., Honorary Ophthalmic Surgeon to the Queen's Jubilee Hospital, London, S.W. **David Sutherland,** M.B., B.S.Edin., Medical Officer to the Worksop Dispensary and Victoria Hospital. **Leslie H. Walsh,** M.D., B.S.Durh., Physician to Bellotts Mineral Water Hospital, Bath.

### XMAS PRESENTS

### A GOOD NURSING BOOK
IS ALWAYS A MOST ACCEPTABLE

### Xmas Present to a Nurse.

### A SELECTION

***Complete Catalogue sent post free on application.***

**HOW TO BECOME A NURSE: How and Where to Train.**

Edited by Sir Henry Burdett, K.C.B.

2/- net, 2/4 post free.

**A HANDBOOK FOR NURSES.**
By J. K. Watson, M.D.
5/- net, 5/4 post free.

**NURSING: ITS THEORY AND PRACTICE.**
By Percy G. Lewis, M.D.
3/6 post free.

**NURSING: ITS PRINCIPLES AND PRACTICE.**
By Isabel Hampton.
7/6 net, 7/10 post free.

**THE NURSE'S PRONOUNCING DICTIONARY OF MEDICAL TERMS AND NURSING TREATMENT.**
By Honnor Morten.
New Edition by Mary I. Burdett.
Cloth, 2/- post free.
In handsome leather gilt, 2/6 net, 2/8 post free.

**MEDICAL GYMNASTICS, including SCHOTT (NAUHEIM) MOVEMENTS.**
By Axel V. Grafstrom, B.Sc., M.D.
2/6 post free.

**ELEMENTARY TREATISE ON THE LIGHT TREATMENT FOR NURSES.**
By James H. Sequeira, M.D. Lond.
2/6 net, 2/9 post free.

**PRACTICAL GUIDE TO SURGICAL BANDAGING AND DRESSINGS.**
By Wm. Johnson Smith, F.R.C.S.
2/- post free.

**NURSING HINTS TO PROBATIONERS ON PRACTICAL WORK.**

By Annesley Voysey.

2/- net, 2/4 post free.

**MEDICAL ELECTRICITY AND THE LIGHT TREATMENT.**
By Sister Kate Neale.
Illustrated. 2/6 net, 2/9 post free.

**ELEMENTARY PHYSIOLOGY FOR NURSES.**
By C. F. Marshall, M.D., B.Sc., F.R.C.S.
Crown 8vo., Illustrated. Cloth, 2/- post free.

**ELEMENTARY ANATOMY AND SURGERY FOR NURSES.**
By William McAdam Eccles, M.S. Lond., M.B., F.R.C.S., L.R.C.P.
2/6 post free.

**A COMPLETE HANDBOOK OF MIDWIFERY.**
By J. K. Watson, M.D. Edin.,
Author of "A Handbook for Nurses."
6/- net, 6/4 post free.

**HOSPITAL SISTERS AND THEIR DUTIES.**
By Eva C. Lückes, Matron, London Hospital.
2/6 post free.

**SURGICAL WARD-WORK AND NURSING.**
By Alexander Miles, M.D. Edin., C.M., F.R.C.S.E.
3/6 net, 3/10 post free.

**ART OF MASSAGE.**

By Mrs. A. Creighton Hale.

6/- post free.

**The Scientific Press, Ltd.**

Publishers of Nursing Text-Books, Charts, and Case-Books of all Descriptions.

28 & 29 SOUTHAMPTON STREET, STRAND, LONDON, W.C.

